# Landscapes for Energy and Wildlife: Conservation Prioritization for Golden Eagles across Large Spatial Scales

**DOI:** 10.1371/journal.pone.0134781

**Published:** 2015-08-11

**Authors:** Jason D. Tack, Bradley C. Fedy

**Affiliations:** 1 Graduate Degree Program in Ecology, Colorado State University, Fort Collins, CO, United States of America; 2 *In cooperation with* Fort Collins Science Center, US Geological Survey, Fort Collins, CO, United States of America; 3 Department of Environment and Resource Studies, University of Waterloo, Waterloo, ON, Canada; University of Lleida, SPAIN

## Abstract

Proactive conservation planning for species requires the identification of important spatial attributes across ecologically relevant scales in a model-based framework. However, it is often difficult to develop predictive models, as the explanatory data required for model development across regional management scales is rarely available. Golden eagles are a large-ranging predator of conservation concern in the United States that may be negatively affected by wind energy development. Thus, identifying landscapes least likely to pose conflict between eagles and wind development via shared space prior to development will be critical for conserving populations in the face of imposing development. We used publically available data on golden eagle nests to generate predictive models of golden eagle nesting sites in Wyoming, USA, using a suite of environmental and anthropogenic variables. By overlaying predictive models of golden eagle nesting habitat with wind energy resource maps, we highlight areas of potential conflict among eagle nesting habitat and wind development. However, our results suggest that wind potential and the relative probability of golden eagle nesting are not necessarily spatially correlated. Indeed, the majority of our sample frame includes areas with disparate predictions between suitable nesting habitat and potential for developing wind energy resources. Map predictions cannot replace on-the-ground monitoring for potential risk of wind turbines on wildlife populations, though they provide industry and managers a useful framework to first assess potential development.

## Introduction

The increasing energy demands of a growing human population pose one of the greatest threats to conserving wildlife populations and their habitats globally [1]. Impacts from energy development can negatively affect wildlife through a suite of direct and indirect mechanisms including habitat loss and fragmentation [2,3], increased mortality [4], spread of invasive species [5], noise pollution [6], and environmental contaminants [7]. The negative impacts associated with these processes can have synergistic effects, and the associated risks to wildlife may also be heightened by catastrophic events. The footprint of current energy development is extensive, and will continue to encroach on wildlife habitats. World energy consumption is expected to rise by >25% by 2030, with the highest growth rates of energy supplies coming from renewable sources [8]. Developing sources of renewable energy pose a paradoxical challenge to wildlife conservation practitioners. Extracting more energy from renewable sources will curb carbon emissions and potentially slow global climate change to protect the future of wildlife populations and their habitats. However, the infrastructure required for developing and maintaining renewable and traditional energy sources often occurs in disparate areas [9]. Therefore, renewable energy development has the potential to impact wildlife populations and their habitats in some of the largest intact landscapes that remain outside of areas traditionally developed for energy exploitation.

Wind energy is a potentially important source of renewable energy globally. In the United States, the Department of Energy established a benchmark of generating 20% of the U.S. electric supply with wind energy by 2030 (http://www.20percentwind.org). This goal will require a dramatic increase in the number of wind turbines throughout the U.S, and the potential effects of large-scale wind energy development on wildlife are not well understood [10]. Thus, guidelines for selecting landscapes to minimize the potentially adverse impacts of wind energy on wildlife are a research priority [11]. Proactively identifying areas for resource development with limited potential impact to wildlife is a promising approach to facilitating energy development while maintaining viable wildlife populations across landscapes [12,13, 14].

Golden eagles (*Aquila chrysaetos*) are a widely distributed raptor of conservation concern in North America [15]. While many raptor species are potentially impacted by increases in wind turbine development [16], golden eagles are a focal species for conservation planning in the United States due, in part, to federal protection they receive under the Bald and Golden Eagle Protection Act (1963). Wind development projects can displace raptors from otherwise suitable habitat, and are a significant source of mortality when placed in areas with high raptor concentrations [17]. For example, Smallwood and Thelander [18] estimated approximately one golden eagle mortality from collisions with wind turbines per 8.7MW of energy produced annually. This level of mortality is of particular concern for long-lived vertebrates, such as eagles, because even a relatively minor increase in adult mortality (3–5%) can lead to significant population declines [19].

Wyoming is emblematic of the challenge to balance wildlife conservation and natural resource development. Wyoming is among the top ten energy producers globally with >100,000 producing oil and gas wells and 400 million tons of coal produced annually (http://www.wma-minelife.com/coal/coalfrm/production.htm). Additionally, Wyoming is among the top five U.S. states in generating electricity from wind power (www.awea.org). The energy-based economy in Wyoming will likely experience continued growth with some of the world’s largest deposits of oil and gas reserves [20], and potential undeveloped wind energy [13].

Our primary objective was to develop a landscape-level planning tool for golden eagle conservation to be used by resource managers and industry prior to the development of renewable energy. Specifically, we aimed to 1) identify golden eagle nesting sites from available data across Wyoming, 2) develop predictive spatial models of golden eagle nesting occurrence, and 3) identify areas of potential conflict and opportunities for golden eagle conservation in the face of expanding wind energy development. Overlays of predictive nesting habitat maps with maps of potential for wind development explicitly delineated areas of potential opportunity for conservation (high quality habitat, low energy potential), and areas posing conflict between development and nesting habitat (high quality habitat, high energy potential).

## Materials and Methods

### Study area

Our sampled population included golden eagle nesting locations across Wyoming, USA. Wyoming encompasses 253,300km^2^ of predominately sagebrush steppe habitat at the junction of the Great Plains and Wyoming Basin ecosystems, with intermittent regions covered by the Rocky Mountains. Land tenure in Wyoming is a mixture of private (44%), federal including the U.S. Bureau of Land Management (BLM; 28%) and Forest Service (14%), and state (6%) ownership. Predominant land uses in Wyoming include cattle grazing, tillage agriculture, and oil and gas energy production.

### Data collection

We requested records from state, federal, non-government, and private entities that collect golden eagle nest data in Wyoming. Access to non-proprietary data resulted in 11,709 records of golden eagle nests between 1974 and 2010. The majority of records were collected between 2000 and 2010 (57%). Records were primarily contributed by the BLM (51%), Wyoming Game and Fish Department (WGFD; 38%), and Thunder Basin National Grasslands (9%). These entities collected location data using a variety of survey methods; including probability-based sampling surveys, mitigation surveys in response to development requests on public lands, and opportunistic sightings. Nest searches included ground-based and aerial methods. We compiled all location data, date of observation, nest status, and source of data. Once the data were compiled, we screened data for consistency in nomenclature and locational accuracy. Any concerns with the data were addressed with the original data managers or censored if irreconcilable problems existed (e.g. uncertainty regarding nest status). The minimum information required for a data record to be included in our analyses was: 1) certainty that the location represented a golden eagle nest (i.e., identification to species level), 2) accurate location data (<120m accuracy), and 3) the year of nesting. Most data had information that could be used to determine the status of the nest following established nomenclature [21] as: active (nests in which eggs have been laid), occupied (a nest with adult presence or strong sign of presence), and inactive (a nest with no apparent recent use or adult presence).

### Delineating the sample unit

The compiled dataset contained records that had considerable redundancy, both within and across nesting seasons. Golden eagle pairs can maintain upwards of 14 nests in a territory [15], and it is likely that groups of nearby nests represent single nesting pairs. Spacing between golden eagle territories (cluster of nests maintained by one pair) varies from 0.8km in southwest Idaho [22] to 44.7km in Quebec [23]. Thus, using all nest records in the full dataset for analyses would likely result in pseudoreplication [24] by including multiple nests within a single territory. We generated an algorithm that identified and reduced spatially dependent clusters of nests to a single nest site based on a hierarchy by year and nest status, while enforcing a minimum spacing between nest sites of 3km, the mean distance between occupied nests across 12 areas in Wyoming [15]. This algorithm minimized underrepresentation of true nest sites available in the sample without proliferating pseudoreplication by treating all nests in the database as independent. To identify nest sites, we first created a data frame of all known nest locations and neighboring nests within 3km. Starting with the most recent year, the algorithm identified each cluster of active and occupied nests to select the nest site with the highest activity level (e.g., ‘active’ trumped ‘occupied’). All nests within 3km of the identified used nest site in that year would then become associated ‘alternate’ nests, and the algorithm would continue to the subsequent year until no records remained to be classified. If there was >1 record with the same year and status in a cluster (i.e. two nests in 2010 classified as active), then one record was chosen randomly to represent the nest site.

### Regional models

Regional variation in habitat availability can confound habitat selection models if not considered explicitly [25]. Landscape features relevant to golden eagle ecology can vary widely across Wyoming, so we developed two regional models to minimize landscape heterogeneity. The North American Commission of Environmental Cooperation (NACEC) Level II Ecoregional Assessment identifies five distinct ecoregions in Wyoming: The Southern and Middle Rockies, Northwestern Great Plains, High Plains, and the Wyoming Basin [26]. Our aim was to build distinct models for each NACEC level II ecoregion (hereafter, ecoregion), though the majority of golden eagle nest data were contained within the Wyoming Basin (WYB) and Northwestern Great Plains (NWGP) regions. Other regions had insufficient data (<30 used nest sites) to estimate RSFs and were censored from analyses. We focused our analyses on the NWGP and WYB regions in the state, which encompassed roughly 2/3 of Wyoming and contained 95% of available nest data.

### Defining availability

Defining an available sample influences the inference derived from habitat selection models [27] and should be conducted at a spatial scale that matches the hierarchical ordering of habitat selection for the sampled unit [28]. We constrained random points to within the Wyoming GAP vertebrate primary and secondary distribution for golden eagle, ensuring random points were within habitat potentially suitable for golden eagle nesting [29]. We saturated the available landscape with available points at 3km spacing [30]. To assign time-specific covariates to available sites, we first calculated the distribution of years represented in nest sites, and randomly assigned a year to each available location based on the proportion of nest sites within that year for each region. This allowed for a similar distribution of time-stamped covariates to be appended to all points because available samples were temporally-varying at the same frequency as nest sites in each region.

### Scale selection

Measuring biotic and abiotic resources at spatial scales relevant to the ecology of a focal species is critical in understanding patterns of habitat selection [31]. Golden eagles demonstrate hierarchical selection for nest sites by choosing suitable cliffs or tall trees [15] locally, that are within a landscape of reliable prey base, and terrain conducive to hunting [32]. Thus, we measured predictor variables at a 200m radius around the nest to capture local-scale attributes associated with nest placement. We also measured predictor variables using a home-range estimate of 3km [33], and 1 and 5km radii to test hypotheses that golden eagles select for habitat at smaller and larger spatial scales.

### Candidate predictor variables

We conducted a literature review and consulted experts to develop hypotheses about environmental and anthropogenic features that influence nest site selection by golden eagles. To test hypotheses, spatial predictor variables had to be available across both the NWGP and WYB regions in Wyoming. For several relevant candidate predictors, complete spatial data were unavailable and for these variables we used surrogate measures that were spatially complete. Furthermore, many spatial data layers were indirectly derived from models and have associated measurement errors (Table 1).

**Table 1:** List and description of spatial variables hypothesized to influence selection of nests by golden eagles. Subscript denotes if multiple scales, quadratic terms, means and standard deviations, or if temporal lag effects of variables were modeled.

Variable	Description
ag_s_	Proportion of tillage agriculture
cliff_s_	Proportion of cliff habitat
fo_s_	Proportion of flat and open habitat (Theobald 2007)
st_s_	Proportion of steep habitat (Theobald 2007)
sl_s_	Proportion of sloped habitat (Theobald 2007)
ndvi_s,q_	Normalized difference vegetation index averaged between 2004 and 2007
treed_s_	Proportion of deciduous and coniferous (non-riparian) tree habitat
r13_s_	Proportion of primary road classes
elev_q_	Digital elevation model of elevation at 30m resolution
ppt4_q,t_	April precipitation
tmin4_q,t_	April mean minimum temperature
tmax4_q,t_	April max minimum temperature
herb_ms_	Estimate of continuous herbaceous cover at 30m resolution
sage_ms_	Estimate of continuous sagebrush cover at 30m resolution
shrh_ms_	Estimate of shrub height averaged at 30m resolution
shrb_ms_	Estimate of continuous cover of all shrubs at 30m resolution
countsofmales_t_	Count of greater sage-grouse males on leks in 5km moving window
countsofleks_t_	Number of active greater sage-grouse leks in 5km moving window

s variable modeled from moving window of scales 200m, 1-, 3-, and 5km.

ms calculated value at each moving window scale, and mean and standard deviation at each scale.

q variable modeled with quadratic term.

t temporally varying covariate modeled with current year, and 1 year lagged effect.

Prey abundance and availability were identified as the most important components of habitat selection by golden eagles during the breeding season [33,34]. Golden eagle prey predominately on Leporids in the North American intermountain West, which comprise up to 70% of their diet during the breeding season [35]. There are no spatial data related to the abundance of Leporids in Wyoming, though recent evidence suggests a strong temporal and spatial correlation between the abundance of cottontail rabbits (*Sylvilagus* sp.) and another prey item of golden eagles, greater sage-grouse (*Centrocercus urophasianus*; hereafter, sage-grouse [36]). Sage-grouse leks (communal breeding grounds) are mapped across Wyoming and almost completely (99%) contained within the golden eagle distribution across the state [29]. Each lek has an associated annual male count that serves as an index to abundance [36,37]. We summed the number of active sage-grouse leks (leks with ≥1 male counted in the most recent two years of observation), for a temporally-varying covariate describing the presence of a lek. We used male counts on leks as an index to abundance, generating year specific layers for the regional variation in sage-grouse lek numbers at the largest scale (5km). To build layers we used the maximum male count within years, and when male count data were missing we used the most recent count for generating layers.

Large scale covariates related to climate and primary productivity may also covary with the abundance of golden eagle prey. We extracted annual estimates of precipitation, and minimum and maximum temperature data from PRISM for June (www.prism.oregonstate.edu). We extracted the year specific estimate to each observation with a quadratic term for precipitation, and also included a one year lagged term by appending data from the previous year of an observation. An index of primary productivity was derived from Normalized Difference Vegetation Index (NDVI) from Moderate Resolution Imaging Spectroradiometer (MODIS) data. We averaged NDVI estimates between a typically wet (2007) and dry (2004) year, and calculated mean and standard deviation of neighborhood values at scales larger than 200m.

Golden eagles typically nest in mid-elevation cliffs [35,38], though they also use ponderosa pine (*Pinus ponderosa*) and Douglas fir (*Pseudotsuga menziesii*) in forested habitats of Wyoming (Bryan Bedrosian, Craighead Beringia South, personal communication). Because analyses were constrained to sagebrush and grassland habitats of NWGP and WYB, we hypothesized that eagles would prefer areas of strong topographic relief locally [39]. Using a 10m National Elevation Dataset we extracted elevation data, and generated topographic indices to describe cliffs, and other landforms. Using the directional landforms tool in Landscape Connectivity and Pattern tools for ArcGIS within a 90m window, we identified flat and open areas, slopes, and steep areas. We also included a covariate for elevation with and without a quadratic term. We classified any pixel with a value <2400m (upper bounds of golden eagle nests) with a change in slope >15 degrees as cliff feature. Pixels in the DEM were classified as a cliff or non-cliff cell based on whether they met the topographic conditions, and the proportion of identified cliff pixels was calculated across all spatial scales.

High quality foraging habitat near nest sites is vital to raising young, and golden eagles typically choose undisturbed sagebrush-steppe habitats with little topographic relief to hunt prey [15,39]. We used data that estimated the percent cover of herbaceous vegetation, sagebrush, and shrub coverage, as well as shrub height at 30m resolution [40]. We calculated the mean and standard deviation of each habitat metric at each spatial scale to estimate landscape heterogeneity which may be related to higher prey populations. Because golden eagles largely avoid forested habitat while foraging [15], we hypothesized nest sites would have a negative association with proportion of forested areas at large scales. To capture forested habitat, we reclassified LANDFIRE existing vegetation cover as forested and non-forested, classifying forested habitat as all non-riparian treed vegetation types (www.landfire.gov). Anthropogenic features relevant to golden eagle ecology that were spatially available across Wyoming, included roads, tillage agriculture, and oil and gas wells. We hypothesized that golden eagles would avoid agriculture at all scales [33,39,41,42,43], and quantified the prevalence of agriculture as the proportion agricultural land within each scale. Data were interpreted from National High Altitude Program (NHAP) color infrared aerial photography or collected with GPS units. Wyoming Water Resources Division provided data on irrigated agricultural lands that we used with a non-irrigated agricultural lands data source, maintained by Wyoming Geographic Information Science Center (WYGISC, http://www.uwyo.edu/wygisc), and a University of Montana irrigated land layer.

Infrastructure associated with oil and gas development is a pervasive feature in Wyoming sage-steppe habitats and includes transmission lines, well pads, roads, and compressor stations. Federal land management agencies require wildlife surveys prior to development. Thus, most survey efforts for golden eagle nests occurred within close proximity of oil and gas developments. Spatial survey bias can have important impacts on model interpretation in habitat suitability studies [44]. The intention of selecting a background group of available sites is to provide a sample of the set of conditions available to the species within the area of interest. If the surveys are not representative of the sampled distribution—in our case, most of Wyoming—then there will likely be bias within the data. Temporal predictor variables of nests and wells may address the potential for biased relationships between nest site selection and well pad density, but sampling of golden eagle nests is still non-random with respect to the energy landscape. In fact, >90% (78% within 3km, 40% within 1km, and 6% within 200m) of nest territories in our dataset are within 5km of an active well underscoring the potential for sampling bias. Thus, we did not include oil and gas well metrics or roads, often associated with energy development, implicitly in our analyses.

We examined if the sampling bias associated with oil and gas development pervaded to covariates in final models by calculating pairwise correlation coefficients between each covariate in predictive models and a metric for oil and gas development among nest sites and available locations. To obtain a measure of oil and gas development, we calculated the number of producing wells prior to nesting dates within each spatial scale around nest sites, using the average date nesting of 23 May for all available sites and nest sites with missing date information. Choosing the best fit scale similar to analyses above, we used the resulting metric for each region for correlation calculations. We determined that covariates used to predict nest-sites would not be biased by the sampling scheme if 1) covariates used in final models and oil and gas measures were weakly correlated (|r|<0.3), and 2) correlations among variables at available sites were not systematically larger than those at used sites. All spatial layers were processed in ArcGIS Desktop V.10.1 (http://www.erdas.com) and retained or resampled to 30m raster data.

### Model development

We developed resource selection functions (RSFs) with a use-availability framework to estimate the relative contribution of predictor variables to nest-site selection by golden eagle [45]. Our aim was to select the best fit term among related variables in the candidate set to use for multivariate modeling. This included choosing a best fit spatial scale among variables, determining whether standard deviation added to model parsimony, and deciding if a quadratic term was appropriate. We compared Akaike Information Criteria (AIC) values among models with the same descriptive variable to determine the best fit spatial scale, and compared AIC values of all models to that of a null logistic regression model. Quadratic terms were competed against the same untransformed univariate models. Once the best fit term was selected from each variable, we examined the correlation structure among variables using pairwise Pearson’s correlation coefficients among all variables. Highly correlated variables (|r|≥0.60) were not included in the same multivariate model. When two variables were highly correlated, we selected the variable with the lowest univariate AIC score for use in multivariate models. This resulted in a suite of variables for each region that was included in the model set for RSF models. We used resulting variables to fit regional global multivariate models for map-based predictions.

### Model evaluation

Using a model evaluation technique developed for RSFs with a use-availability design by Boyce et al. [46], we portioned data using k-fold cross validation with five folds for each region. We iteratively fit global models for each set of training folds, and calculated the area-adjusted observed number of observations falling into 10 binned RSF classes. We calculated the Spearman rank correlation between the RSF score and the area-adjusted frequency of validation points for each of the five folds and the mean area adjusted frequency across folds. High correlation values between RSF scores and area-adjusted frequencies suggest a model that is good at predicting the occurrence of golden eagle nests [45].

### Forecasting wind development risk

We converted NREL wind power class (WPC) maps from polygon to raster data, resampling to 30m pixels to match GIS layers used in RSF development (http://www.nrel.gov/gis/wind.html). NREL maps provide an estimate of the annual average wind resources at 50m, delineated into 7 wind power classes (1 lowest WPC, 7 highest WPC), so we similarly reclassified regional RSF maps into 7 geometric bins. We combined WPC maps with RSF maps and generated a raster with each pair of RSF and WPC categories for a total of 49 unique cell values. For each region, we calculated the area of each RSF and WPC combination, and tabulated the number of nest sites and commercial wind turbines (as of 2009) within each category.

## Results

### Data and model results

**Fig 1:**
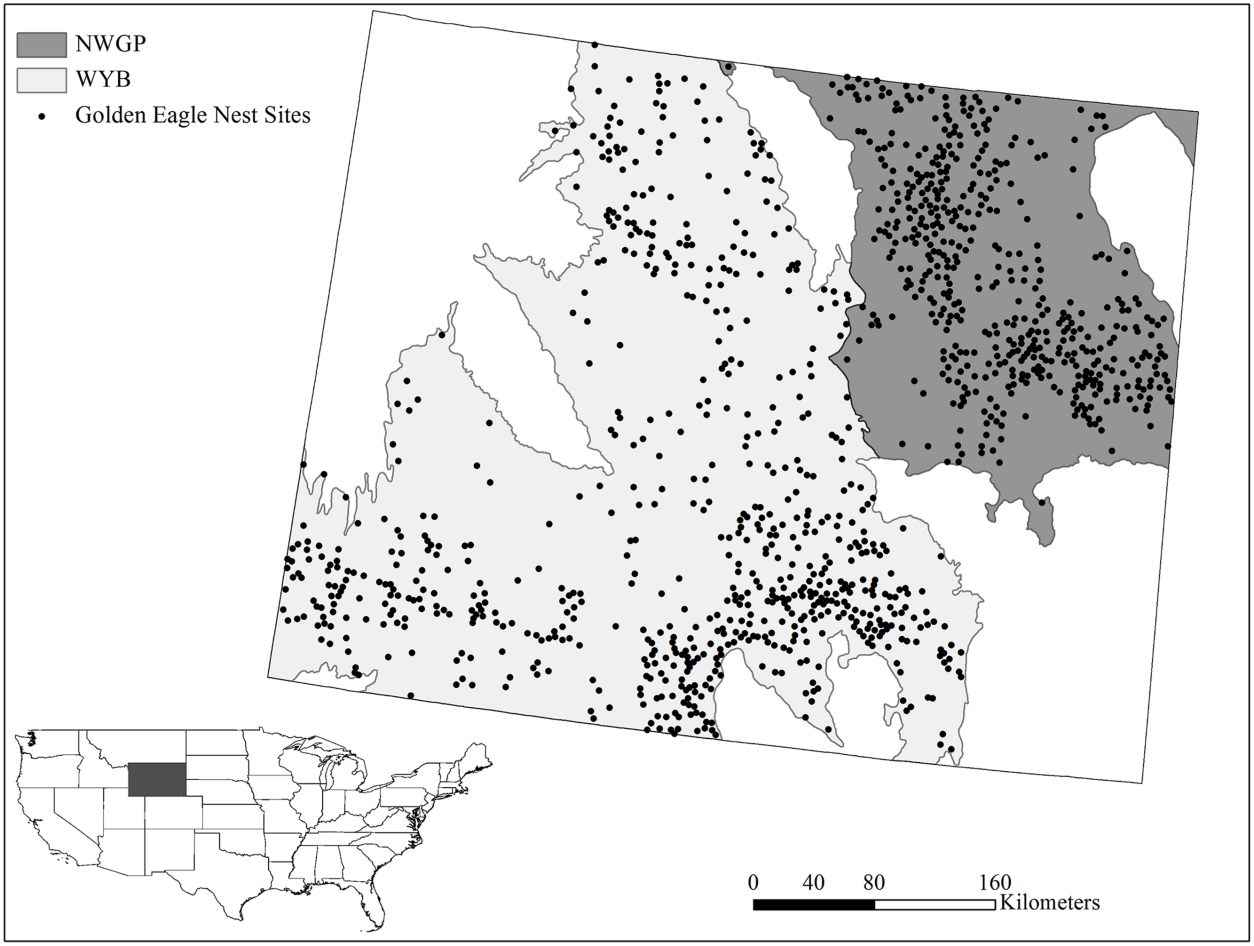
North American Commission of Environmental Cooperation (NACEC) level II ecoregions Northwest Great Plains (NWGP; dark gray), and Wyoming Basin (WYB; light gray) portions of Wyoming, USA. Reducing nest site data to remove redundant and clustered data produced 1,176 total nest sites, 483 in the NWGP and 693 in the WYB.

Removing possible redundant nests with our hierarchical selection algorithm based on nest spacing, observation year, and activity status (active, occupied, and inactive) identified 1,176 nest sites. Of the 1,176 nest sites 483 were located in the Northwest Great Plains region (NWGP) and 693 were in the Wyoming Basin region (WYB; Fig 1). The oldest nest site data were from 1974 and 40% of nest sites were from 2000–2010. Saturating each region systematically with available samples while enforcing 3km spacing between all data point resulted in 4,158 available samples in the NWGP, and 11,053 in the NWGP.

All variables considered in the regional models contained at least one term that fit better than a null model. In the NWGP, best-fit scales associated with variables where either the smallest (200m) scale for cliffs and steep landscapes; or the largest possible scale (5km; Table 2), with AIC values increasing or decreasing monotonically towards each selected scale (unpublished data). A standard deviation term improved the AIC score for all sagebrush metrics and an inclusion of a one year lagged effect better modeled the influence of sage-grouse leks and precipitation on nest-site selection. Best fit multi-scaled variables in the WYB were similar to NWGP models except for NDVI and sloped areas (Table 2).

**Table 2:** Best fit univariate term among competing variables in the Northwest Great Plains (NWGP) and Wyoming Basin (WYB), and coefficient estimate. Asterisks denote correlated variables removed from multivariate RSF models.

Variable	NWGP	WYB
ag	5km (-0.28)	200m (-0.41)
cliff	200m (0.25)	200m (0.60)
ndvi	5km^2^ (-0.59, 0.06)	1km^2^ (0.01, -0.18)*
treed	5km (-0.72)	5km (-0.16)*
flat/open	200m (0.24)	200m (-0.34)*
sloped	5km (0.16)	1km (0.29)
steep	200m (0.18)*	200m (0.41)*
herb	5km_m,sd_ (-0.11, -0.36)*	5km_m,sd_ (-0.30, -0.13)
sage	5km_m,sd_ (0.23, -0.47)	5km_m,sd_ (0.08, -0.17)
shrh	5km_m,sd_ (-0.15, -0.56)*	5km_m,sd_ (0.08–0.12)*
shrb	5km_m,sd_ (-0.05, -0.17)*	5km_m_ (-0.18)
sg lek count	lag (0.29)	cur (0.14)
sg malecount	lag (0.25)*	lag (-0.06)*
tmin	cur^2^ (0.21, -0.19)	cur^2^ (0.19, -0.15)
tmax	cur^2^ (0.14, -0.10)*	cur^2^ (0.16, -0.16)*
ppt	lag^2^ (-0.08, -0.15)	cur^2^ (-0.10, -0.06)
elev	(-0.49, -0.18)	(-0.06, -0.18)

m—mean;

sd-standard deviation;

^2^-quadratic term;

cur—current year; lag– 1 year lagged

* Correlated variable removed for inclusion in multivariate model

Each regional suite of variables contained several predictors that were highly correlated (r>|0.60|; Table 2). Remaining uncorrelated variables shared the direction of selection (positive or negative) across regions used for global models (Table 2). Variables used in global models were not correlated (|r|<0.24) with the number of producing oil and gas wells within 5km (Table 3).

**Table 3:** Pairwise correlation values between variables used in global RSF models and best fit term associated with oil and gas development (producing wells within 5km).

NWGP			WYB		
Variable	Available	Used	Variable	Available	Used
ag 5km	0.14	0.16	ag 200m	0.01	-0.04
cliff 200m	-0.03	0.16	cliff 200m	-0.02	-0.03
ndvi 5km	-0.02	0.08	slope 1km	-0.03	-0.14
ndvi 5km^2^	-0.11	-0.14	herb 5km m	-0.03	-0.05
treed 5km	-0.12	0.00	herb 5km sd	0.00	0.03
flat/open 200m	-0.01	-0.05	sage 5km m	-0.04	-0.21
slope 5km	0.00	0.23	sage 5km sd	0.00	-0.12
sage 5km m	-0.03	0.01	shrb 5km m	-0.04	-0.10
sage 5km sd	-0.02	0.05	lek count	-0.03	-0.08
lek count lag	-0.03	0.02	tmin	-0.05	-0.09
Tmin	-0.07	-0.20	tmin^2^	0.04	-0.07
tmin^2^	-0.06	-0.06	ppt	0.02	0.04
ppt lag	0.04	0.14	ppt^2^	0.02	-0.05
ppt lag^2^	-0.08	-0.12	elev	-0.03	-0.13
Elev	0.00	-0.09	elev^2^	0.01	0.03
elev^2^	-0.14	-0.03			

**Fig 2:**
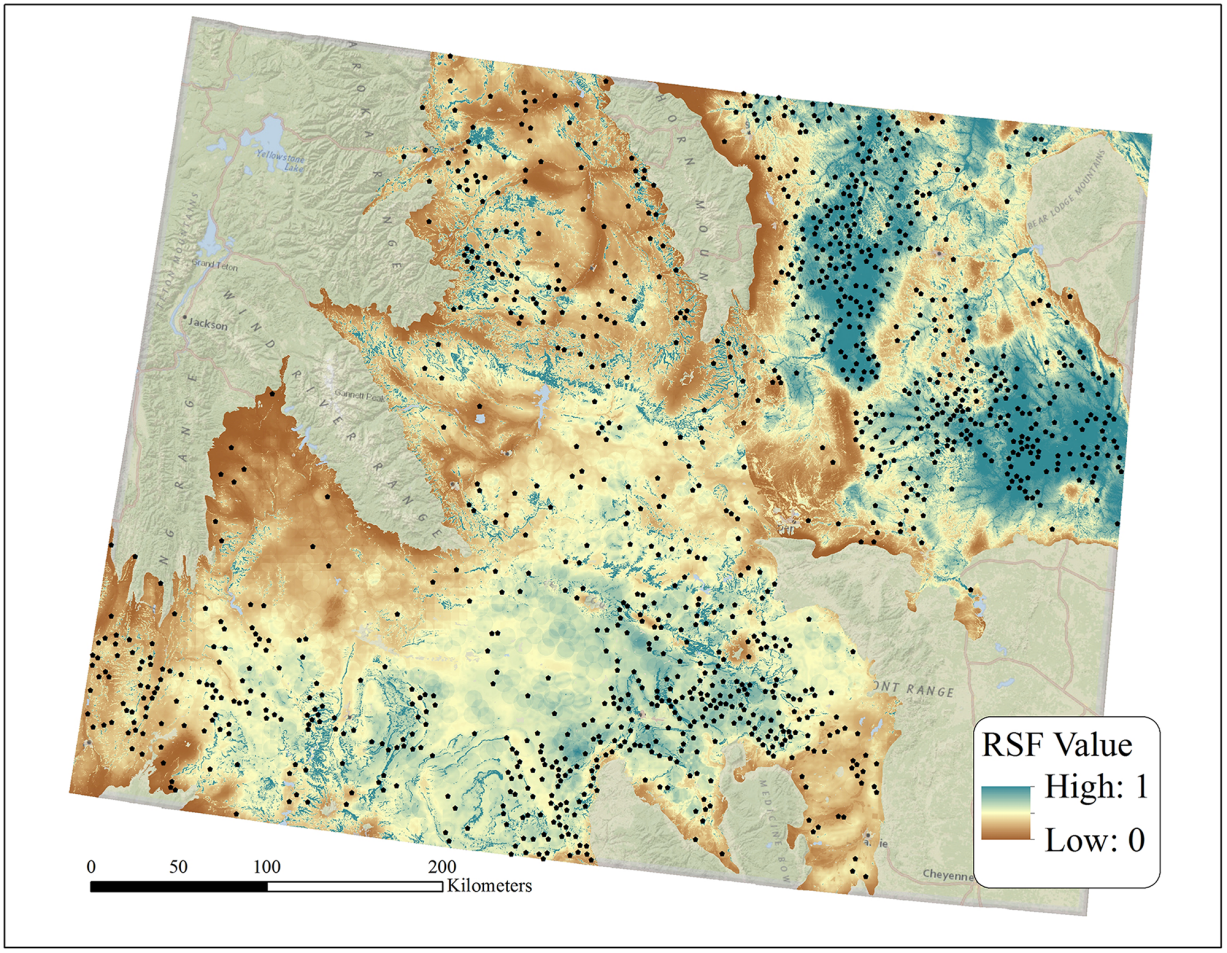
Resource selection function (RSF) probability grids across the Northwest Great Plains (NWGP) and Wyoming Basin (WYB) regions in Wyoming, USA. RSF values represent the probability proportion to use of golden eagle nest site. Predictions are based on a global model for each region.

Global models for each region contained 15 covariates representing topographic indices, prey density, land use, climate, and vegetation (Table 4; Fig 2). We removed an agricultural predictor variable from the NWGP regional model because the coefficient estimate changed significantly in direction and magnitude from the univariate estimate, suggesting variable instability [47]. Among variables occurring in each regional model, elevation and temperature differed in the direction for which they influenced nest site selection (Table 4). Spearman rank correlation values between the area-adjusted frequency of validation points and RSF bin across the five folds ranged from 0.86–0.95 in the NWGP, and 0.72–0.96 in the WYB, while averages from across folds were high in the NWGP (1.0) and WYB (0.952).

**Table 4:** Coefficient estimates and standard errors for global RSF models in the Northwest Great Plains (NWGP) and the Wyoming Basin (WYB).

NWGP			WYB		
Variable	β	SE	Variable	β	SE
cliff 200m	0.38	0.042	ag 200m	-0.07	0.087
ndvi 5km	-0.53	0.083	cliff 200m	0.64	0.028
ndvi 5km^2^	-0.02	0.072	slope 1km	0.11	0.052
treed 5km	-0.53	0.146	herb 5km m	-0.41	0.079
flat/open 200m	0.38	0.051	herb 5km sd	0.03	0.089
slope 5km	0.29	0.064	sage 5km m	-0.01	0.071
sage 5km m	-0.02	0.065	sage 5km sd	0.00	0.070
sage 5km sd	-0.40	0.066	shrb 5km	-0.20	0.081
lek count lag	0.23	0.045	lek count	0.18	0.030
tmin	-0.07	0.069	tmin	0.11	0.057
tmin^2^	-0.10	0.049	tmin^2^	-0.11	0.037
ppt lag	-0.15	0.065	ppt	-0.11	0.057
ppt lag^2^	-0.10	0.050	ppt^2^	-0.07	0.037
elev	-0.75	0.079	elev	0.21	0.086
elev^2^	0.06	0.055	elev^2^	0.01	0.052

**Fig 3:**
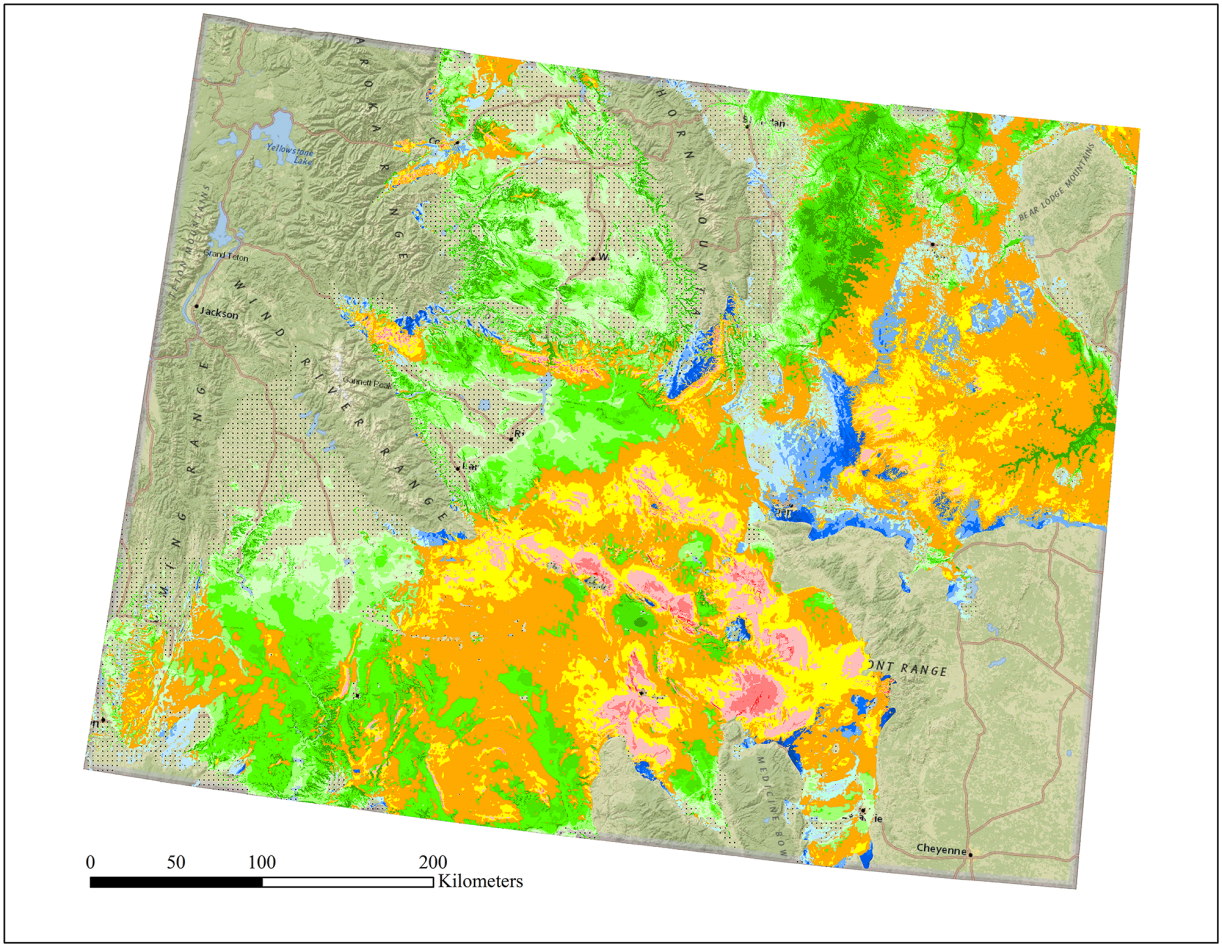
Spatial delineation of overlay between seven NREL wind power classes (WPC; 1-low wind value, 7-high wind value) and regional resource selection function maps grouped into seven geometric bins (see Fig 4 for color legend). Hatched areas are predicted low value for golden eagle nesting and wind development.

**Fig 4:**
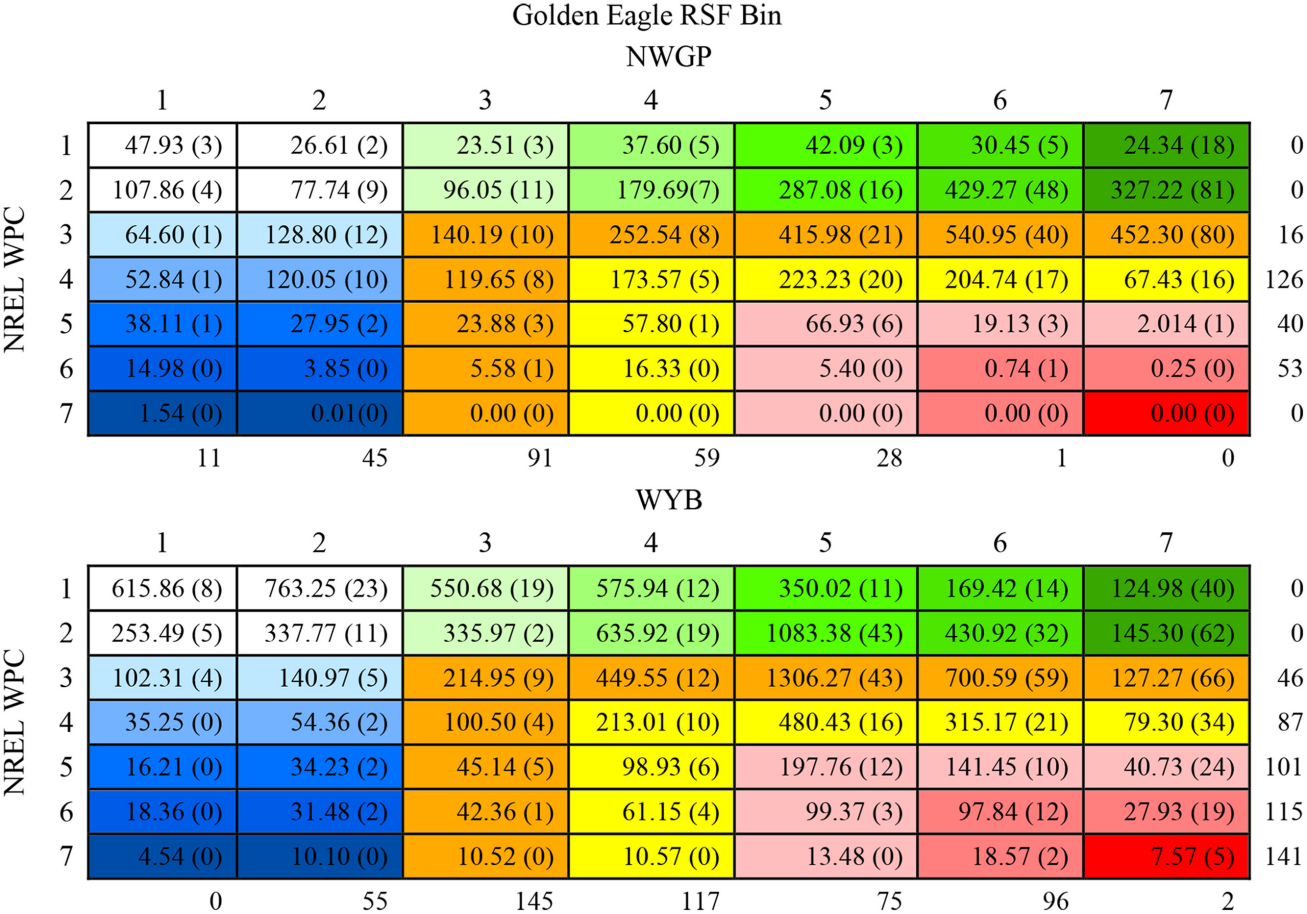
Area (km^2^) and the known number of nests (in parentheses) found overlapping cells between golden eagle RSF and NREL wind power class (WPC) map in the Northwest Great Plains (NWGP) and the Wyoming Basin (WYB). Values on outside of tables represent the number of wind turbines in each category as of 2009. Cell colors correspond to map in Fig 3.

Areas identified as moderate risk (orange and yellow colors; Figs 3 and 4) made up the greatest portion of the study area when overlaying NREL WPC maps with reclassified golden eagle RSF maps (Figs 3 and 4). Cells considered the highest risk (RSF 7 and WPC 7) represented the smallest area in both regions (Figs 3 and 4), containing no observed nests in the NWGP and 5 in the WYB. Across regions the lowest three WPC (1–3) contained 75% of the known nest sites, while the highest 3 WPC contained only 10% of nests (Fig 4). The number of wind turbines within each WPC increased monotonically in the WYB; in the NWGP, WPC 4 contained the most existing turbines (Fig 4). The most wind turbines across regions occurred in RSF bins 3–4, with only 2 turbines occurring in the highest or lowest RSF bin (Fig 4).

## Discussion

We modeled breeding habitat selection for a wide-ranging predator across large spatial scales—over twice the land area of Austria. Our models performed well, despite the generalist nature of golden eagles, likely due to the large number of samples and availability of high-quality GIS data across our study areas. Processes influencing nest site selection in golden eagles are dynamic and complex, including land forms, vegetation, and a temporally-variant climate and prey base. Cliff features at local scales were important predictors in both regions. Selection for flat and open areas in the NWGP could appear at odds with selection for cliff; however, the landform metric for flat and open areas was summarized at 90m, in contrast to the 30m resolution of the cliff metric. The apparent disparity between these metrics likely reflects the selection for areas of sharp relief within flat and open areas.

Eagles demonstrated slight preference for less vegetated areas, demonstrated by a negative association with the Normalized Difference Vegetation Index (NDVI) in the NWGP at the largest scale, and negative coefficient estimates for herbaceous cover at large scales in the WYB. Higher NDVI values and herbaceous cover may result in higher densities of primary consumers including multiple prey species for golden eagle, though they may also obscure visibility of prey. Lower NDVI values could also distinguish sage steppe from grassland habitats, as golden eagles preferentially selected for sagebrush cover in large contiguous tracts at large scales.

Prey abundance and distribution is paramount in explaining space use of predatory species. Though spatial data on prey abundance and distribution is rarely available across landscape scales. Researchers have used models of prey distribution, including coefficients drawn from RSF models, to explain attributes of predator habitat use [48,49]. However, our research uses direct measures of prey distribution and abundance to estimate the influence on predator habitat selection. Models suggest golden eagles selected nest sites within landscapes containing greater numbers of sage-grouse leks. This preference may likely capture the spatial and temporal correlation between sage-grouse and cottontail rabbits [36], a primary prey resource of golden eagles [15, 35].

Infrastructure associated with oil and gas development may influence golden eagle nest-site selection; however, the potential sampling bias within energy landscapes rendered these variables inappropriate for nesting models. This bias did not pervade to the covariates we sampled, as the best estimate of oil and gas was only weakly correlated with variables included in regional models. Investigating the impacts of development requires mechanistic studies beyond the scope of our data, and should include measures of habitat use and overall fitness of individuals. Human disturbance may decrease the probability of golden eagles occupying territories [50] and many wildlife species in Wyoming including sage-grouse [51], antelope [52], mule deer [53], and grassland birds [6] alter habitat use in response to oil and gas development. However, the avoidance documented in other wildlife species may not apply to raptors. Indeed, raptors may selectively use anthropogenic features associated with oil and gas development such as power lines and roads for hunting. In fact, ferruginous hawks (*Buteo regalis*) have been documented nesting on drilling equipment in tree-sparse prairies [54]. Though a lack of avoidance does not necessarily indicate a lack of impact, and it is possible that features selected in a human-modified landscape may have unintended consequences on overall fitness of raptors [55].

Despite good model performance, the nest data used to develop our models were not collected according to an optimum sampling design when considering the state of Wyoming as a single study site. Most of the data were collected to address localized research and management needs regarding golden eagle nesting ecology. When designing a research study, investigators should carefully consider the optimum sampling design to address the questions of interest prior to data collection [56]. Yet design-based studies in ecology are currently rare at large spatial and temporal scales, and investigators often must combine data from multiple sources. Careful consideration of sampling design and species ecology can help ensure valid conclusions are drawn from the data. For example, the first step in designing a research study involves defining a sampling frame and unit. We defined the sampling frame as the known distribution of golden eagles within Wyoming, though identifying the sampling unit required more consideration. We developed a hierarchical approach to parsing available data to define a biologically meaningful sampling unit. Our approach focused on our specific study objectives and incorporated species behavior, sampling time frames, data quality, and the spatial distribution of records. We recommend the development of similar approaches when working with datasets collated from multiple surveys with varying study objectives.

NREL wind power class (WPC) and RSF map overlays demonstrated 1) Wyoming landscapes are dominated by areas of moderate suitability to nesting golden eagles and wind development, 2) high quality eagle habitat and high WPC values have minimal overlap, 3) nests tend to occur in lower WPC, and 4) existing turbines tend to occur in lower RSF values. It is important to note that potential wind resources are but one factor leading to the likely installation of wind turbines, demonstrated by the non-monotonic relationship between existing turbines and WPC. Various social, political, infrastructure, and environmental factors likely converge in the decision-making process among stakeholders. Yet energy development is ultimately linked to available resources, and NREL maps provide a powerful broad-scale tool with utility in applied research [13,57,58] for resource managers and industry. Viewed in total, we found that high quality golden eagle nesting habitat and areas of high value for wind energy installations are largely disparate. Yet our models did classify over 700km^2^ as containing the highest quality golden eagle nesting habitat (RSF 5–7) in the three highest WPC. These “risky” areas encompassed 98 known golden eagle nest sites and roughly 1/3 (250) of the commercial turbines in the study area.

Our risk maps provide a biological basis for helping to guide the siting of wind development at local and landscape levels. Our predicted maps contain 30m resolution, and thus have the ability to provide guidance for site-level placement of turbines within existing permitted fields, though we do not suggest that models displayed as maps should replace empirical on-site monitoring. In particular, non-breeding habitat-use should be evaluated when making siting decisions. Anecdotal evidence suggests that most eagle turbine strikes occur during spring breeding months, though mortalities are seasonally ubiquitous [59,60]. Identifying winter raptor concentration areas, juvenile (non-breeding) dispersal areas, and understanding migratory pathways, will be important contributions of applied research towards eagle conservation.

## Conclusions

Minimizing golden eagle mortality and displacement is the major goal in research efforts to identify areas of high species use prior to wind development. Currently, most preconstruction risk assessments are based upon site-level monitoring prior to wind turbine placement. Typically, assessments use abundance indices and assume linear relationships with future mortality (i.e., risk scales directly with observed bird counts). However, studies have found that local abundance is often not correlated with mortality at wind farms [61], and that environmental impact assessments based upon bird counts at sites do not share a relationship with recorded mortality following construction [62]. Large scale, spatially explicit, and empirically driven habitat use models such as those presented here may be better predictors of mortality risk for certain species. For example, Carrete et al. [63] found that models predicting the distribution and aggregation (e.g., breeding colonies and roost sites) of griffon vultures (*Gyps fulvus*) across large extents had a positive and linear relationship with mortalities at wind farms. Indeed, large scale spatially-explicit models near aggregation areas (e.g., nests) far outperformed pre-construction counts for estimating mortality risk [63]. Our overlays of wind potential and probability of nest selection have taken this a step further in an attempt to identify high risk areas prior to development. We suggest the consideration of our models in concert with site-level multi-season data to help inform development concerned with minimizing impacts to golden eagles in Wyoming. The greatest strength of our products to managers lies in the ability to proactively target areas for conservation where the biological value is highest and the energy development risk is minimal. Used in concert with additional species-level habitat maps purveying risk across Wyoming, including sage-grouse, managers in Wyoming have a scientifically-defensible toolbox to help achieve multiple-species conservation at a landscape level.
